# Translation and validation of the Chinese version of the quality of life radiation therapy instrument and the head & neck module (QOL-RTI/H&N)

**DOI:** 10.1186/1477-7525-12-51

**Published:** 2014-04-14

**Authors:** Xin-lin Chen, Zhen-wen Qiu, Mo-fa Gu, Yong Su, Li-zhi Liu, Yan Liu, Chuan-wei Mo, Qian Xu, Juan Sun, Dong-hai Li

**Affiliations:** 1 Department of Biostatistics and Preventive Medicine, School of Basic Medical Sciences, Guangzhou University of Chinese Medicine, Guangzhou, Guangdong province, China; 2 Department of biostatistics, School of Public Health, Southern Medical University, Guangzhou, Guangdong province, China; 3 The First Affiliated Hospital, Guangzhou University of Chinese Medicine, Guangzhou, Guangdong province, China; 4 Department of Radiation Oncology, State Key Laboratory of Oncology in South China, Cancer Center, Sun Yat-sen University, Guangzhou, Guangdong province, China; 5 Department of Radiology, State Key Laboratory of Oncology in South China, Cancer Center, Sun Yat-sen University, Guangzhou, Guangdong province, China; 6 The First Affiliated Hospital, Guangzhou Medical University, Guangzhou, Guangdong province, China; 7 School of Human Social Science, Guangzhou University of Chinese Medicine, Guangzhou, Guangdong province, China

**Keywords:** Head and neck cancer, Quality of Life, QOL-RTI/H&N, Translation, Validation

## Abstract

**Background:**

To translate and validate the Chinese version of the Quality Of Life Radiation Therapy Instrument and the Head & Neck Module (QOL-RTI/H&N), a disease-specific scale to measure quality of life (QOL) for patients with head and neck cancer (HNC) who received radiotherapy.

**Methods:**

The QOL-RTI/H&N was translated and validated according to the standard process: a translation and back-translation procedure, pilot testing and a validation study. HNC patients were enrolled from the Cancer Center of Sun Yat-sen University and assessed using the QOL-RTI/H&N, QLQ-C30 and QLQ-H&N35. Reliability (internal consistency reliability, split-half reliability and test-retest reliability), validity (content validity, construct validity, criterion validity and discriminant validity), and responsiveness analysis were performed to evaluate the psychometric characteristics of the QOL-RTI/H&N.

**Results:**

A total of 238 patients (99.2%) completed the questionnaire. Item RTI23 had 16.0% missing data. Other items had low percentages of missing data (0.4% or 0.8%) or no missing data. The average time to finish the scale was 9.8 minutes. Cronbach's alpha of the domains ranged from 0.41 to 0.77. The split-half reliability coefficients ranged from 0.43 to 0.77. All of the intra-class correlation coefficients were equal to or greater than 0.8. All of the item-own domain correlation coefficients were greater than those of the item-other domain. Confirmatory factor analysis showed that Comparative Fit Index, Normed Fit Index and Non-Normed Fit Index were equal to 1.00. Root Mean Square Error of Approximation was 0.01, with 90% CI (0.00, 0.10). The domain scores of the QOL-RTI/H&N were significantly correlated with those of the QLQ-C30 or QLQ-H&N3. All domain scores of patients in different radiotherapy stages were statistically significant (*P* < 0.05), apart from the speech domain.

****Conclusions**:**

The Chinese version of the QOL-RTI/H&N is a valid, reliable and responsive scale to measure QOL in HNC patients and can be used to assess the effects of radiotherapy treatment on these patients.

## Background

Head and neck cancer (HNC) is the sixth most common cancer worldwide [1]. According to data from the International Agency for Research on Cancer, the number of HNC patients (including lip, oral cavity, esophagus, larynx, nasopharynx and other pharynx) was estimated to be more than 1,140,000, with incidence and mortality rates at 15.1 and 10.0 per 100,000, respectively, in 2012 [2]. Nasopharyngeal cancer is one of the most prevalent HNCs in China, and is much more prevalent than in the rest of the world [3].

Radiotherapy (RT) is the main treatment for HNC patients. However, RT treatment not only kills the tumor cells, but also injures the normal local tissues [4,5]. HNC patients receiving RT treatment are subjected to numerous of adverse effects, such as dry mouth, thick saliva, and difficulties in opening the mouth, swallowing and speaking. All of these adverse effects drastically reduce their quality of life (QOL). Therefore, patient-based outcome measures, such as QOL and Patient-Reported Outcomes (PROs), have attracted more and more attention in recent years [6].

The common scales to evaluate QOL in HNC patients include the EORTC Quality of Life Questionnaire - Core Questionnaire (QLQ-C30) and the Head and Neck Cancer Module (QLQ-H&N35) [7-11], the Functional Assessment of Cancer Therapy - General (FACT-G) and the Head & Neck Scale (FACT-H&N) [12,13], the Quality Of Life Radiation Therapy Instrument and Head & Neck Module (QOL-RTI/H&N) [14,15], the Head and Neck Radiotherapy Questionnaire (HNRQ) [16], the University of Washington Quality Of Life questionnaire (UWQOL) [17], the Quality of Life Instrument for Head and Neck Cancer (QL-H&N) [18], the Quality of Life Questionnaire for advanced HNC patients (QLQ) [19], and the University of Michigan Head and Neck Quality Of Life (HNQOL) [20]. Among these scales, only the HNRQ, QLQ-H&N35 and QOL-RTI/H&N were specifically designed to evaluate the QOL of HNC patients receiving radiotherapy [14-16]. The HNRQ only focuses on the side effects of RT treatment and was not recommended for independent use [16]. The QLQ-H&N35 has been translated into Chinese, and has been reported as a valid, reliable and responsive scale [21]. The QOL-RTI/H&N was developed to evaluate QOL in HNC patients after RT treatment by researchers in the Division of Radiation Oncology of the University of South Florida. The QOL-RTI/H&N already had the versions in English, Japanese, and German, as well as a version developed specifically for Hong Kong [14,15,22-24]. All versions showed good validity and reliability.

The aim of this study was to develop the Chinese (Mainland China) version of the QOL-RTI/H&N. We translated and back-translated the English version, and adapted it linguistically and culturally in China. We documented the translation, back-translation, cultural adaptation and psychometric testing of the scale.

## Methods

### Translation and back-translation procedure

The English version of the QOL-RTI/H&N was translated according to the standard process for translating instruments [25-28]. Permission to use the QOL-RTI/H&N was obtained from the original authors. Two bilingual (Chinese and English) native speakers translated the original version into Chinese independently. The translation coordinator (one of the authors) compared the two Chinese versions and reconciled any differences. At last, the team, including the two translators, three radiotherapy doctors, two radiotherapy nurses, two QOL experts and four H&N cancer patients, compiled the Chinese version and chose the most appropriate wording for clarity and similarity to the original. The final Chinese version was formed after the team discussed culturally problematic issues.

The Chinese version was translated back into English by two separate translators independently, neither of whom had previously seen the scale. The two back-translated versions were also coordinated and discussed by the team. The changing of sentences and wordings were approved to obtain the results of the back-translated version.

### Pilot testing

Pilot testing was used to assess cultural adaptation and content validity of the Chinese version of the QOL-RTI/H&N. All of the items were tested in a convenience sample of 10 HNC patients from different educational levels. The time taken to complete the QOL-RTI/H&N was less than 12 min. Problematic items were revised according to the comments of the patients. Cultural adaptation was summarized and content validity was assessed.

The pilot testing found that most items in the Chinese version and the original version had similar meanings. Only a few were revised. For example, HNC patients reported that Item 17 (IRT17) “support from God” was difficult to understand. So Item IRT17 was revised as “I get support from faith or religion, for example, Buddhism, Taoism, etc.” The patients reported that they were embarrassed to answer Item IRT23 “My sexual activity is satisfactory”. However, no appropriate sentence could be found to replace it, so the original sentence remained.

### Validation study

A cross-sectional study was conducted to measure the reliability, validity and responsiveness of the scale. The patients pathologically diagnosed with HNC in the Cancer Center of Sun Yat-sen University were enrolled from July 1st to September 15th in 2012. The patients who had other types of cancer and those who were illiterate were excluded. The written permission and approval for this study was obtained from the committee board in the Cancer Center of Sun Yat-sen University.

The investigators were medical graduate students who received specific training. They explained the aim of our study to the HNC patients. After obtaining informed consent from the patients, they gave each patient a questionnaire to complete by self-administration, including a socio-demographic sheet, and the QOL-RTI/H&N, QLQ-C30 and QLQ-H&N35. The socio-demographic sheet covered age, gender, marital status, monthly income, dialect, current work status, types of disease, other chronic disease, as well as the time of first radiotherapy. According to the time of first radiotherapy, RT stages were divided into 5 groups: before RT, under RT, ≤1 year after RT, ~5 years after RT, and >5 years after RT. If patients did not fully understand the meaning of the items, the investigators explained them clearly or used other understandable words with the same meaning. At last, the investigators made efforts to help the patients complete the questionnaire.

All of the HNC patients were required to fill in the QOL-RTI/H&N within 24–48 hours. The patients who were newly diagnosed with HNC for the first time were also required to finish the scale within 28 ± 2 days of RT treatment.

### Questionnaires

The QOL-RTI/H&N consists of the QOL-RTI and the H&N module. The QOL-RTI is a general scale with 24 items, assessing components of function, emotion, family/socio-economics (family for short) and general (overall) QOL [14]. The H&N module consists of domains related to pain, appearance, speech, swallowing and chewing (swallow for short), saliva and mucous (saliva for short), taste, and cough [15]. Each item is rated on a 0–10 Likert type scale. The scores for all items in each domain are summed and average to yield a single score. A higher score indicates a better QOL.

### Statistical analyses

The analyses consisted of the description of the characteristics, assessment of item quality, reliability analysis, validity analysis and responsiveness analysis. Most of the statistical analyses were done with Windows SPSS 17.0 (SPSS Inc, Chicago, IL). Confirmatory factor analysis (CFA) was conducted using Lisrel software (Version 8.7) [29].

Item quality was assessed using mean, standard deviation (SD), missing data, lowest (floor) and highest (ceiling) scores. Reliability analysis included internal consistency reliability, split-half reliability, and test-retest reliability. Internal consistency reliability was evaluated using Cronbach's alpha value. Split-half reliability was assessed by Pearson's correlation coefficients between two halves of the items. Test-retest reliability coefficients were calculated by the intra-class correlation coefficient (ICC), along with 95% confidence interval (CI) of the two scores within 24–48 hours. The short test-retest time interval (24 ~ 48 hours) was chosen for the following reasons. (1) All of the HNC patients enrolled in the study were being treated with RT. The treatment had an obvious influence on QOL, particularly when the time interval was long. (2) Marx *et al*. reported no significant differences for the test-retest reliability of the two time intervals (2 days and 2 weeks) [30].

Validity analysis included content validity, construct validity, criterion validity, and discriminant validity. Content validity was compared using the item-own domains (the item and its own domain) and the item-other domains (the item and other domains) correlation coefficients. If the correlation coefficient of item-own domains was greater than that of item-other domains, then the item had good content validity. CFA was used to measure the construct validity and assess how well the data fit the theoretical model [31-33]. Comparative Fit Index (CFI), Normed Fit Index (NFI), Non-normed Fit Index (NNFI), Root Mean Square Error of Approximation (RMSEA) and its 90% CI were all calculated. Values of CFI, NFI and NNFI equal to or greater than 0.9 were considered to have an adequate fit, and those near to 1 as having a good fit [34]. RMSEA less than 0.05 indicated a good fit [35,36]. Criterion validity was obtained by calculating the Pearson correlation coefficients of the domain scores between the QOL-RTI/H&N and the QLQ-C30 or QLQ-H&N35. Discriminant validity was measured by comparing the QOL of patients in different RT stages, which were analyzed by analysis of variance (ANOVA). If the QOL of the patients in different RT stages was significantly different, then the scale had good discriminant validity.

Paired samples t-test was used to analyze the QOL changes over time for the patients receiving RT treatment the first time. If the QOL had improved or decreased significantly after treatment, it meant that the scale had good responsiveness. Effect size was calculated as the change in scores divided by the SD of the baseline score. Effect size greater than 0.5 was considered to be clinically significant [37].

## Results

A total of 240 patients were enrolled in the study. Two patients refused to complete the questionnaire. Finally, 238 (99.2%) patients completed the questionnaire. Four patients completed the assessment with the help of the investigators, because they did not fully understand the meaning of the questionnaire. The age ranged from 21.7 to 78.8, with a mean age of 46.5. There were 176 male patients and 62 females. 95.4% of the patients were married. 64.3% of the patients were Cantonese. 71.4% were patients with nasopharyngeal carcinoma (Table 1).

**Table 1:** **Characteristics of the patients (*n*** **= 238)**

	**Number of patients (%)**
Year, mean ± SD (range)	46.5 ± 10.4 (21.7, 78.8)
Gender	
Male	176 (73.9)
Female	62 (26.1)
Marital status	
Unmarried	11 (4.6)
Married	227 (95.4)
Monthly income	
≤2000 Yuan	87 (36.6)
~5000 Yuan	119 (50.0)
>5000 Yuan	18 (7.6)
Unclear	14 (5.9)
Dialect	
Cantonese	153 (64.3)
Hakka	45 (18.9)
Chaoshan	13 (5.5)
Others	27 (11.3)
Other chronic disease	
No	189 (79.4)
Yes	49 (20.6)
Tumor site	
Nasopharynx	170 (71.4)
Non-nasopharynx	68 (28.6)
RT	
Before	50 (21.0)
Under	40 (16.8)
≤ 1 year after	57 (23.9)
~5 years after	50 (21.0)
>5 years after	41 (17.2)

*Non-nasopharynx* included oral, oropharyngeal, hypopharyngeal, salivary gland, laryngeal and paranasal cancer. *RT*: radiotherapy.

The scores of all the items ranged from 0 to 10 (Table 2). Item RTI17 scored the highest (9.41), while item RTI18 scored the lowest (2.44). Item RTI23 had 16.0% missing data. Items RTI9, RTI12, RTI14, RTI16, RTI25, RTI29 and RTI30 had low missing data (0.4% or 0.8%). Other items did not have any missing data. Item RTI18 showed 47.1% of responses at floor. Item RTI17, RTI39 and RTI16 showed 72.7%, 67.2%, and 50.4% of responses at ceiling, respectively.

**Table 2:** **Mean, SD, missing data, floor and ceiling scores of each item (*n*** **= 238)**

**Item**	**Mean**	**SD**	**Missing(%)**	**Floor(%)**	**Ceiling(%)**	**Item**	**Mean**	**SD**	**Missing(%)**	**Floor(%)**	**Ceiling(%)**
RTI1	6.55	3.04	0.0	3.4	19.3	RTI21	7.23	2.39	0.0	0.4	26.1
RTI2	6.68	2.94	0.0	0.4	21.4	RTI22	6.97	2.94	0.0	0.4	25.2
RTI3	5.97	3.20	0.0	1.7	16.4	RTI23	5.84	2.75	16.0	3.4	11.8
RTI4	7.67	3.15	0.0	5.5	39.1	RTI24	6.79	2.74	0.0	1.3	16.4
RTI5	5.28	3.08	0.0	5.5	12.2	RTI25	6.53	1.93	0.8	1.3	5.9
RTI6	6.44	3.32	0.0	7.6	20.6	RTI26	7.41	3.15	0.0	4.2	34.0
RTI7	3.99	3.35	0.0	18.1	7.1	RTI27	6.95	3.36	0.0	6.7	33.2
RTI8	5.31	2.75	0.0	6.3	7.1	RTI28	3.79	3.50	0.0	10.1	13.4
RTI9	5.81	3.44	0.4	6.3	21.8	RTI29	3.55	3.31	0.4	15.5	9.7
RTI10	7.78	2.22	0.0	0.4	30.7	RTI30	4.44	3.55	0.4	8.8	18.1
RTI11	7.99	2.33	0.0	1.3	31.9	RTI31	6.00	3.53	0.0	5.5	22.7
RTI12	8.16	2.30	0.4	1.3	37.8	RTI32	6.71	3.16	0.0	5.5	23.5
RTI13	5.13	3.36	0.0	9.7	15.5	RTI33	7.78	2.83	0.0	3.4	33.6
RTI14	5.59	3.61	0.8	12.6	22.7	RTI34	7.65	2.75	0.0	2.5	36.1
RTI15	7.01	2.37	0.0	0.8	22.3	RTI35	5.59	3.16	0.0	7.1	14.3
RTI16	8.71	1.87	0.4	1.3	50.4	RTI36	7.47	2.48	0.0	2.5	26.1
RTI17	9.41	1.34	0.0	0.8	72.7	RTI37	6.82	3.24	0.0	5.9	28.2
RTI18	2.44	3.20	0.0	47.1	7.1	RTI38	5.68	3.21	0.0	5.9	18.1
RTI19	7.35	3.20	0.0	2.9	37.8	RTI39	8.95	2.32	0.0	2.1	67.2
RTI20	7.14	2.05	0.0	0.8	14.3						

*Mean score*: 0 (worst QOL) to 10 (best QOL). *RTI1*: the first item of the QOL-RTI/H&N scale, etc. *SD*: standard deviation.

The mean score of the saliva domain was 3.93, and mean scores of other domains were higher than 6.00 (Table 3). Cronbach's alpha values of all domains were between 0.41 (family) and 0.77 (pain). The split-half coefficients of all domains ranged from 0.43 (family) to 0.77 (pain). The test-retest coefficients of all domains were equal to or greater than 0.8 (0.80 ~ 0.94). All item-own domain correlation coefficients exceeded 0.4, which were greater than those of the item-other domain.

**Table 3:** **Descriptive statistics and reliability of the QOL-RTI/H&N (*n*** **= 238)**

**Domain**	**No. of items**	**Range of score**	**Mean ± SD**	***a^***	**Split-half coefficient**	**ICC(95% CI)**	**Item-own domain correlation**	**Item-other domain correlation**
Function	9	(0.6, 10.0)	6.50 ± 1.83	0.77	0.72	0.82(0.70, 0.89)	0.50 ~ 0.71	0.02 ~ 0.44
Emotion	7	(1.7, 10.0)	6.22 ± 1.38	0.49	0.53	0.90(0.84, 0.94)	0.44 ~ 0.64	0.086 ~ 0.41
Family	6	(4.0, 10.0)	6.83 ± 1.24	0.41	0.43	0.80(0.67, 0.88)	0.42 ~ 0.65	0.04 ~ 0.32
General	3	(1.7, 10.0)	6.97 ± 1.70	0.71	0.68	0.88(0.79, 0.93)	0.79 ~ 0.83	0.11 ~ 0.55
Pain	2	(0.0, 10.0)	7.18 ± 2.93	0.77	0.77	0.87(0.78, 0.92)	0.89 ~ 0.91	0.17 ~ 0.44
Swallow	5	(1.2, 10.0)	6.90 ± 1.91	0.67	0.65	0.93(0.88, 0.96)	0.50 ~ 0.73	0.02 ~ 0.44
Saliva	3	(0.0, 10.0)	3.93 ± 2.82	0.74	0.70	0.89(0.82, 0.94)	0.79 ~ 0.84	0.08 ~ 0.41
Appearance	1	(0.0, 10.0)	6.71 ± 3.16	NA	NA	0.87(0.75, 0.93)	1.000	0.14 ~ 0.40
Speech	1	(0.0, 10.0)	7.78 ± 2.83	NA	NA	0.89(0.80, 0.94)	1.000	0.06 ~ 0.15
Taste	1	(0.0, 10.0)	6.00 ± 3.53	NA	NA	0.94(0.89, 0.96)	1.000	0.08 ~ 0.41
Cough	1	(0.0, 10.0)	7.65 ± 2.75	NA	NA	0.87(0.76, 0.93)	1.000	0.15 ~ 0.42

*a^*Cronbach's alpha value. *NA* meant no value, due to one single item.

**Figure 1:**
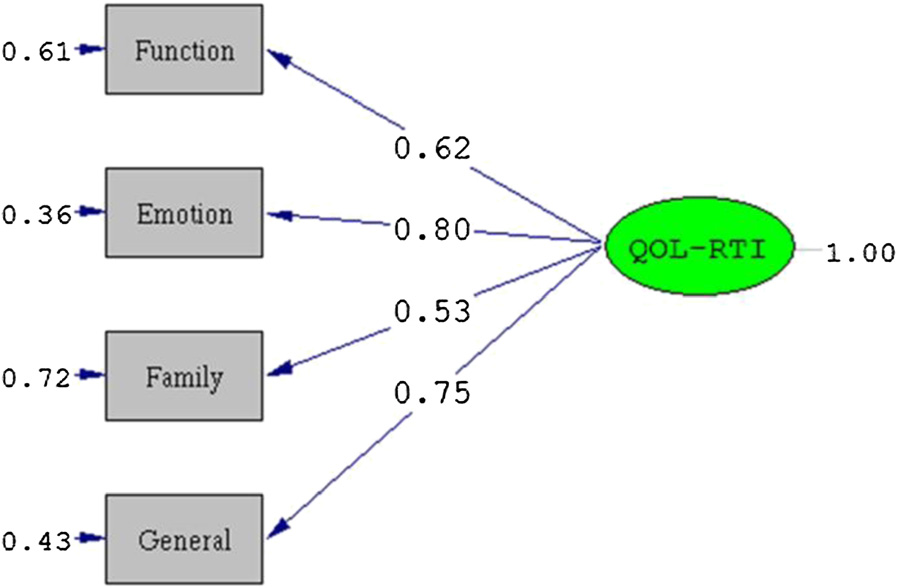
The CFA structure diagram of the QOL-RTI scale.

**Figure 2:**
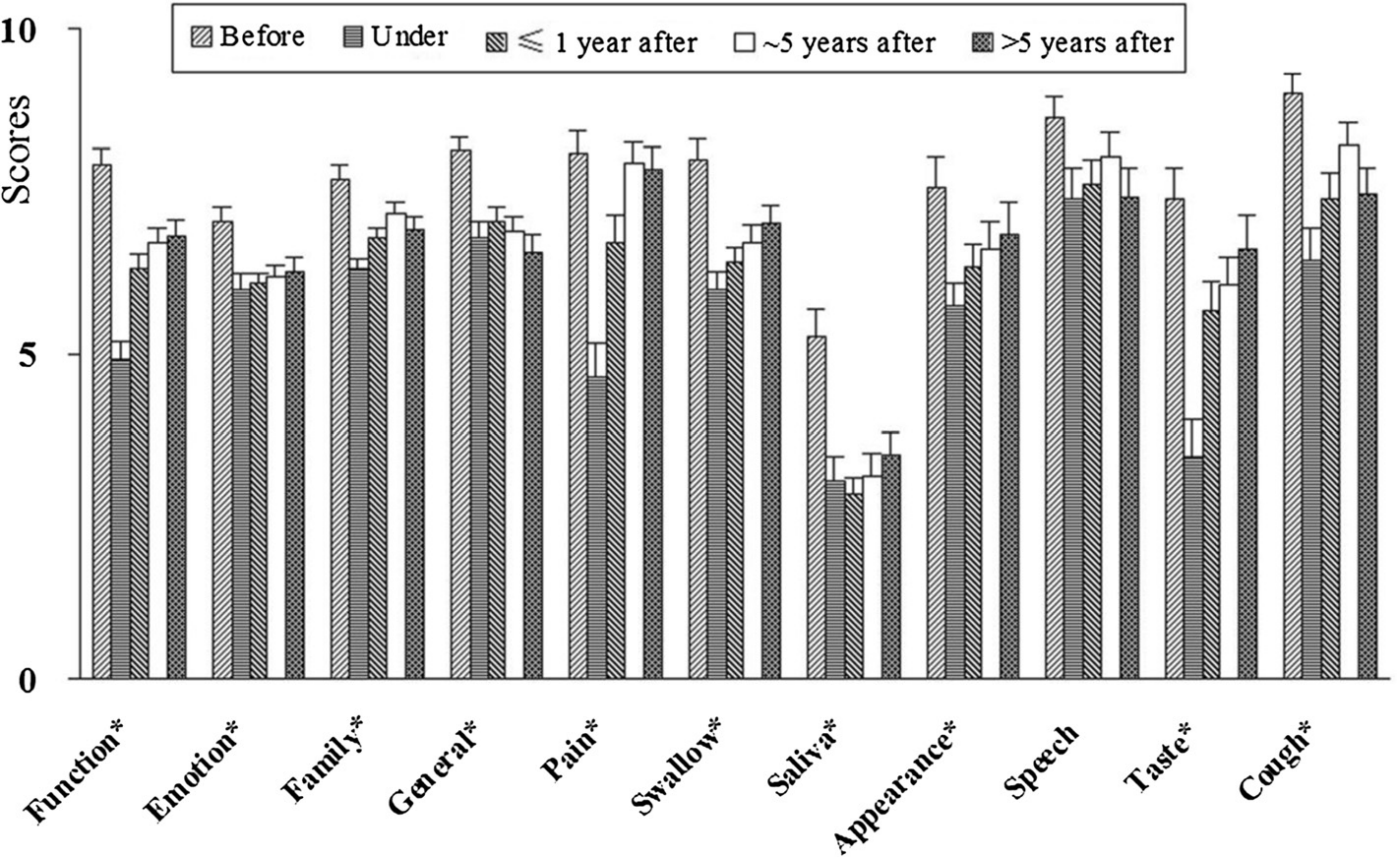
The domain scores in different RT stages (mean ± SE, *: significantly different).

The results of the CFA showed that normal theory weighted least squares Chi-Square of the model was equal to 2.11 (*P* = 0.35 > 0.05). CFI, NFI and NNFI were equal to 1.00. RMSEA was 0.01, with 90% CI (0.0, 0.10) (Figures 1 and 2).

Criterion validity was tested by comparing the QOL-RTI/H&N with the QLQ-C30 and QLQ-H&N35. There were positive correlation coefficients (0.28 to 0.45) between QOL-RTI domain scores and corresponding scores of the QLQ-C30 (Table 4). The pain, swallow, speech, and cough domains in the H&N module had significantly negative correlations with those of the QLQ-H&N35 (*P* <0.05).

**Table 4:** **Criterion validity of the QOL-RTI/H&N (*n*** **= 238)**

**QOL-RTI/H&N**	**QLQ-C30, QLQ-H&N35**	**Correlation coefficient**
Function	PF of QLQ-C30	0.34*
Emotion	EF of QLQ-C30	0.32*
Family	SF of QLQ-C30	0.28*
General	QL of QLQ-C30	0.45*
Pain	Pain of QLQ-H&N35	-0.56*
Swallow	Swallow of QLQ-H&N35	-0.32*
Speech	Speech of QLQ-H&N35	-0.35*
Cough	Cough of QLQ-H&N35	-0.53*

*PF*: Physical functioning; *EF*: Emotional functioning; *SF*: Social functioning; *QL*: Global health. *meant *P* < 0.05. The Saliva, appearance, and taste domains did not have criterion validity.

All domain scores of patients in different RT stages were significantly different (*P* <0.05), except for those of the speech domain (Table 5, Figure 2). The patients who were receiving RT treatment (under) generally had the lowest QOL scores, while those who had not received RT treatment (before) had higher QOL scores. No statistically significant differences emerged for any of the QOL domains when examined by gender, tumor site, or other disease (see Additional file 1).

**Table 5:** Domain scores (Mean ± SD) in different RT stages

	**Before (*n*** **= 50)**	**Under (*n*** **= 40)**	≤** 1 year after RT (*n*** **= 57)**	**~5 years after RT (*n*** **= 50)**	**>5 years after RT (*n*** **= 41)**	**P**
Function	7.90 ± 1.70	4.91 ± 1.68	6.31 ± 1.58	6.72 ± 1.54	6.81 ± 1.65	<0.001
Emotion	7.03 ± 1.55	6.00 ± 1.46	6.08 ± 1.27	6.19 ± 1.32	6.27 ± 1.45	0.002
Family	7.69 ± 1.46	6.30 ± 1.04	6.79 ± 1.16	7.16 ± 1.12	6.91 ± 1.27	<0.001
General	8.13 ± 1.56	6.79 ± 1.47	7.04 ± 1.65	6.87 ± 1.71	6.57 ± 1.83	<0.001
Pain	8.08 ± 2.59	4.66 ± 3.25	6.73 ± 3.03	7.93 ± 2.29	7.83 ± 2.33	<0.001
Swallow	8.00 ± 2.12	5.99 ± 1.81	6.41 ± 1.56	6.73 ± 1.80	7.02 ± 1.80	<0.001
Saliva	5.24 ± 3.16	3.07 ± 2.13	2.84 ± 1.93	3.13 ± 2.37	3.44 ± 2.29	<0.001
Appearance	7.56 ± 3.27	5.72 ± 2.31	6.33 ± 2.76	6.62 ± 2.97	6.86 ± 2.92	0.042
Speech	8.62 ± 2.38	7.39 ± 2.96	7.62 ± 2.81	8.04 ± 2.64	7.42 ± 2.81	0.148
Taste	7.40 ± 3.40	3.41 ± 3.54	5.67 ± 3.44	6.06 ± 3.06	6.62 ± 3.20	<0.001
Cough	9.02 ± 1.89	6.44 ± 3.09	7.39 ± 2.87	8.22 ± 2.39	7.46 ± 2.69	<0.001

Fifty patients who had received RT treatment for the first time were enrolled to test the responsiveness of the scale over time. After 28 days of treatment with RT, all domain scores decreased (Table 6). The change in scores for all domains were significantly different, with effect sizes ranging from 0.22 (family domain) to 1.23 (saliva domain). Except for emotion, family, general and speech domains, the effect sizes of other domains were greater than 0.5.

**Table 6:** **Change in scores and effect size after 28 days of treatment (*n*** **= 50)**

	**Baseline score**	**Change scores**^**a**^	**95% CI of Change scores**	**Effect size**^**b**^
Function	7.90 ± 1.70	2.03 ± 0.87	(1.79, 2.27)	1.19
Emotion	7.03 ± 1.55	0.39 ± 0.77	(0.18, 0.60)	0.25
Family	7.69 ± 1.46	0.32 ± 0.65	(0.14, 0.50)	0.22
General	8.13 ± 1.56	0.52 ± 0.58	(0.36, 0.68)	0.33
Pain	8.08 ± 2.59	2.93 ± 1.09	(2.63, 3.23)	1.13
Swallow	8.00 ± 2.12	2.20 ± 0.99	(1.93, 2.47)	1.04
Saliva	5.24 ± 3.16	3.88 ± 1.92	(3.35, 4.41)	1.23
Appearance	7.56 ± 3.27	2.16 ± 0.98	(1.89, 2.43)	0.66
Speech	8.62 ± 2.38	1.00 ± 0.95	(0.74, 1.26)	0.42
Taste	7.40 ± 3.40	3.94 ± 1.66	(3.48, 4.40)	1.16
Cough	9.02 ± 1.89	2.18 ± 0.80	(1.96, 2.40)	1.15

^a^Change scores: -10 (maximum improvement) to +10 (maximum worsening).

^b^Effect size: calculated as change in scores divided by the SD of the baseline score.

The average time to finish the scale was 9.8 ± 4.3 minutes, ranging from 7.2 to 13.6 minutes. Four patients reported that the 0–10 Likert style was difficult for them to understand. They were not able to clearly discriminate the 0–10 Likert style. They preferred the 5-Likert style with an explanation.

## Discussion

With the help of RT treatment, HNC patients survive for a longer time. However, the complications and side effects of RT treatment seriously affect their QOL. The insufficiency of a specific radiotherapy scale measuring the QOL of Chinese HNC patients led us to translate and validate the QOL-RTI/H&N.

The QOL-RTI/H&N had good content validity. The WHOQOL group proposed that QOL incorporates the physical health, psychological state, social relationships, and general relationships of the individual to salient features of the environment, independence and belief [38,39]. The QOL-RTI is structurally made up of function, emotion, family, and general domains, which are the main domains defined by the WHOQOL group. The H&N module consists of 7 symptom domains (pain, appearance, speech, swallowing, saliva, taste, and cough). It includes the most important clinical aspects characterizing specific aspects of QOL for HNC patients, such as “I have pain in my mouth,” “I have pain in my throat,” “I have a normal amount of saliva,” “My saliva is too thick,” and “I can swallow all food without difficulty”. These items could truly reflect the suffering of HNC patients receiving RT treatment. Additionally, all item-own domain correlation coefficients were greater than that of the item-other domain.

The Chinese version of the QOL-RTI/H&N had good reliability (internal consistency reliability, split-half reliability and test-retest reliability). The QOL-RTI/H&N had moderate or high values for Chronbach's alpha (0.41-0.77). The emotion and family domains had the lowest values. This result may stem from some inconsistent items in these domains, such as the appearance and financial items in the emotion domain, and the independence item in the family domain. A similar result was observed in the Hong Kong version of the scale [22]. They reported that Cronbach's alpha values in other domains were greater than 0.7, while those of emotion and family domains were 0.58 and 0.61, respectively. Our results showed that all domains had moderate or high split-half reliability coefficients (0.43-0.77), and had high test-retest reliability coefficients (0.80-0.94), which is consistent with previous studies [15,22,24]. It was reported that test-retest reliability coefficient of the scale was 0.87 for the German version [24], and was 0.92 for the Hong Kong version [22].

The Chinese version of the QOL-RTI/H&N had good validity (construct validity, criterion validity, and discriminant validity), as well. CFI, NFI and NNFI of the QOL-RTI were equal to 1.00, and RMSEA was 0.01, with a 90% CI (0.0, 0.10). All indices indicated good construct validity for the scale. Theoretically similar domains of the QOL-RTI/H&N and the QLQ-C30 or QLQ-H&N35 were significantly related to each other at low or moderate levels. Therefore, the scale had good criterion validity. All domains (except the speech domain) of the QOL-RTI/H&N were shown to be sensitive to discriminate the QOL of HNC patients in different RT stages (significantly different), which suggested the scale had good discriminant validity. None of the domain scores of HNC patients among different genders, other chronic diseases, and tumor sites were significantly different. These results were consistent with the hypothesis. The scale was developed to measure QOL in HNC patients receiving RT treatment. Therefore, it was not significantly associated with gender, other chronic diseases or tumor site.

The sensitivity and responsiveness of the QOL-RTI/H&N were confirmed. The HNC patients suffered numerous symptoms, especially head and neck symptoms after RT treatment, such as pain in the mouth and throat, dry mouth, and speaking difficulties. Therefore, the effect sizes of the function and H&N domains were greater than those of emotion, family, and general domains. Similar decreases in QOL were observed in the English version, the Hong Kong version and the German version of the scale [15,22,24]. The effect sizes of other domains, except for emotion, family, general and speech domains, were greater than 0.5. Therefore, the function domain and the H&N module domains were considered to be sufficiently clinically sensitive to reflect QOL changes in HNC patients who received RT treatment.

In addition, the scale had good operability and acceptability. The response rate of the scale was 99.2% (238/240). Most of the items had complete data. Item IRT23 “My sexual activity is satisfactory” had 16.0% missing data, however. Chinese people usually feel embarrassed to talk about their sexual activity or sexual life. The patients completed the scale in an average time of 9.8 minutes. However, 4 patients said that the 0–10 Likert style scale was difficult to understand.

The study had some limitations. One is that all HNC patients in the study were enrolled from the Cancer Center of Sun Yat-sen University. Further assessment of the QOL-RTI/H&N should be performed with results from multiple centers to confirm the generalizability of the findings. The second constraint is that a short test-retest time interval (24 ~ 48 hours) was allowed in this study. The short time interval may potentially cause the overestimation of test-retest reliability. The third limitation is that only 50 HNC patients were used to test the responsiveness of the QOL-RTI/H&N scale. The responsiveness of the scale should be further assessed in a larger sample of patients.

## Conclusions

The Chinese version of the QOL-RTI/H&N was translated according to the standard process for translating instruments: a translation and back-translation procedure, pilot testing and a validation study. The scale is valid for measuring QOL in HNC patients with good reliability, validity and responsiveness. We recommend the application of the QOL-RTI/H&N for measuring QOL in HNC patients and assessing the effects of RT treatment.

## Abbreviations

QOL-RTI/H&N: Quality of life radiation therapy instrument and the head & neck module; HNC: Head and neck cancer; QOL: Quality of life; RT: Radiotherapy; PROs: Patient-reported outcomes; QLQ-C30: Quality of life questionnaire - core questionnaire; QLQ-H&N35: Quality of life questionnaire head and neck cancer module; CFA: Confirmatory factor analysis; SD: Standard deviation; ICC: Intra-class correlation coefficient; CI: Confidence interval; CFI: Comparative fit index; NFI: Normed fit index; NNFI: Non-normed fit index; RMSEA: Root mean square error of approximation; PF: Physical functioning; EF: Emotional functioning; SF: Social functioning; QL: Global health; α: Cronbach’s alpha.

## Competing interests

The authors declare that they have no competing interests.

## Authors’ contributions

XLC conceived of and designed the study, analyzed the data, drafted and revised the manuscript. ZWQ was involved in the design of the study, interpreted the data and drafted the manuscript. MFG conceived of and designed the study, collected the data, and drafted the manuscript. YS collected the data and discussed the statistical analysis. LZL was involved in the recruitment of patients, discussed statistical analysis and revised the manuscript. YL revised and modified the manuscript. CWM and QX analyzed the data. JS help to draft the manuscript. DHL interpreted the data. All authors read and approved the final manuscript.

## Supplementary Material

Additional file 1 The domain scores in patients with different gender, other disease and tumor site. Click here for file
