# Supplementary material for: Translation and validation of the Chinese version of the quality of life radiation therapy instrument and the head & neck module (QOL-RTI/H&N)
Source: Health Qual Life Outcomes. 2014 Apr 14;12:51. doi: 10.1186/1477-7525-12-51 (PMC4022371; doi:10.1186/1477-7525-12-51)
Supplement: Additional file 1 — The domain scores in patients with different gender, other disease and tumor site. [file 1477-7525-12-51-S1.pdf]

Additional file 1

Table A. the domain scores (Mean ± SD) in patients with different gender, other disease and tumor site

	N	Function	Emotion	Family	General	Pain	Swallow	Saliva	Appearance	Speech	Taste	Cough
Gender												
Male	176	6.66±1.77	6.36±1.44	7.05±1.25	7.20±1.68	7.13±2.92	6.89±1.95	3.53±2.94	6.45±3.21	7.85±2.72	5.87±3.44	7.82±2.78
Female	62	6.38±1.74	6.23±1.26	6.76±1.33	6.91±1.55	7.02±2.54	6.72±1.99	3.60±2.78	7.02±2.94	7.83±2.62	5.97±3.28	7.60±2.63
P		0.283	0.516	0.123	0.235	0.803	0.547	0.875	0.224	0.966	0.839	0.582
Other disease												
Yes	49	6.71±1.88	6.50±1.23	7.08±1.35	7.12±1.62	7.61±2.45	7.04±2.00	3.61±3.08	6.97±2.95	8.28±2.55	6.16±3.49	8.07±2.47
No	189	6.55±1.74	6.27±1.42	6.95±1.26	7.11±1.68	6.97±2.90	6.79±1.95	3.54±2.85	6.50±3.20	7.72±2.72	5.82±3.37	7.68±2.81
P		0.573	0.300	0.517	0.970	0.158	0.438	0.882	0.348	0.209	0.534	0.378
Tumor site												
Nasopharynx	170	6.53±1.71	6.34±1.36	7.00±1.28	7.09±1.63	7.22±2.66	6.73±1.97	3.45±2.87	6.72±3.02	7.87±2.56	5.75±3.36	7.76±2.59
Non-nasopharynx	68	6.73±1.91	6.27±1.48	6.92±1.26	7.17±1.71	6.82±3.19	7.12±1.90	3.80±2.96	6.69±3.45	7.78±3.02	6.26±3.48	7.76±3.10
P		0.432	0.727	0.637	0.736	0.324	0.173	0.398	0.343	0.820	0.289	1.000
